# Supplementary material for: D2FLS-Net: Dual-Stage DEM-guided Fusion Transformer for landslide segmentation
Source: PLoS One. 2025 Nov 26;20(11):e0337412. doi: 10.1371/journal.pone.0337412 (PMC12654934; doi:10.1371/journal.pone.0337412)
Supplement: Attachment — Submitted filename: PONE-D-25-44116.docx [file pone.0337412.s001.docx]

**Ref:** PONE-D-25-44116

**Title:** D2FLS-Net: Dual-Stage DEM-Guided Fusion Transformer for Robust Landslide

Segmentation from Remote Sensing Imagery

This study presents a novel Swin Transformer-based framework (D2FLS-Net) for landslide segmentation, featuring two innovative modules: Dual-Stage DEM-Guided Fusion (DSDF) and Terrain-aware Pixel-wise Adaptive Context Enhancement (T-PACE). The work demonstrates strong technical merits and achieves state-of-the-art performance on two benchmark datasets. The topic would be interesting to readers of *PLOS ONE*. While the study is partly interesting and valuable, There is a large room for improvement in the language, novelty, contents, and figures of the manuscript. I **recommends major revision of** this manuscript for the reasons outlined below:

**Abstract**

(1) Lines 27: module should be deleted
(2) Please add quantitative comparison to DEM fusion baselines in the Abstract, which can enhance the novelty of the manuscript.
(3) The main novelty of the manuscript should be highlighted. Why do authors propose a novel to identify the boundary of landslides? The reasons for developing this study should be clarited.
(4) “remote sensing imaery” and “feature fusion” should be included in Keywords.

**Introduction/Methodology**

(1) Lines 64-71,114-125. The authors should summary the main achievement of existing literature not simply listing study contents.
(2) Main contribution of the study should be described in Conclusion section, not the end of Introdution section.
(3) Reorganize logic of Introduction section. The author should tell readers why developing this study.
(4) Line 313 F1 equation is not found, and the F1 indicator is also not used to evaluate the performance of the model.

**Results and analysis**

(1) The manuscript should elaborate on why two specific fusion stages (Swin-1 and Swin-4) were selected rather than intermediate stages. Provide ablation results testing alternative stage combinations.
(2) 700 samples are too few, which can not fully train deep learning models. From Table 1, the number of weighted coefficient (w), and bias (b) are over 1000. The authors should develop cross-validation strategy to illustrate the model robustness.

**Conclusions:**

(1) This section should tell readers what main contributions of the manuscript is.

**Figures**

(1) The quality of the figures is too poor to recognize texts.
