# Supplementary material for: D2FLS-Net: Dual-Stage DEM-guided Fusion Transformer for landslide segmentation
Source: PLoS One. 2025 Nov 26;20(11):e0337412. doi: 10.1371/journal.pone.0337412 (PMC12654934; doi:10.1371/journal.pone.0337412)
Supplement: Attachment — Submitted filename: Response to Reviewers.docx [file pone.0337412.s003.docx]

PONE-D-25-44116

D2FLS-Net:Dual-Stage DEM-guided Fusion Transformer for landslide segmentation

PLOS ONE

All mentioned line numbers correspond to the new "manuscript" or "Revised Manuscript with Track Changes.docx document under simple markup"

==============================

**Response to Editor’s Specific Comments**

1. Strengthen the positioning of your contribution. Highlight *what is fundamentally new* compared to existing DEM+optical fusion models.

**Response**

Compared with existing DEM and optical fusion methods, which apply channel level fusion, feature fusion, or semantic fusion, our model is designed as a data fusion approach grounded in landslide triggering mechanisms and in the target characteristics of the two major categories of landslides. For so-called new landslides, where the landslide body is clearly distinguishable from the surrounding background, optical data alone can recognize landslides well, and fully introducing DEM data would lead to the Hughes phenomenon (when the feature dimensionality passes a critical threshold, further increases lead to degraded classifier performance). Therefore, we design the early DEM to be integrated in a guiding manner in the form of weights, while mainly relying on the subsequent Terrain aware Pixel wise Adaptive Context Enhancement for boundary segmentation; after a period of time, new landslides may experience vegetation regrowth or long term weathering and erosion and thus form so-called old landslides (which remain necessary to recognize because they are prone to retriggering by earthquakes and rainfall). For such landslides, DEM that contains topographic and geomorphic information becomes particularly important, and at this time the early DEM weights use strong features (primary features) to guide landslide recognition when optical data cannot provide effective segmentation and the loss increases, and in the later stage the interaction and fusion with optical features further increases the active role of DEM. The final model is presented as D2FLS-Net: Dual-Stage DEM-guided Fusion Transformer for landslide segmentation.

**Changes made**

We have re-emphasized the contributions and innovations at the end of the Abstract and Introduction (lines 115–126). We appreciate the reviewer’s comments and have revised the text accordingly with careful respect.

2. Discuss how the model could be scaled or deployed in practice (e.g., pilot projects, global applicability).

**Response**

Since our approach employs a Transformer model, which has already been extensively validated across diverse domains, scalability can be achieved by enlarging the landslide dataset and by increasing the depth and width of the network. For practical deployment, however, it is essential to ensure low latency and energy consumption. This requires techniques such as weight quantization and the conversion of the PyTorch implementation into high-performance formats like TensorRT for accelerated inference.

**Changes made**

The authors discussed future optimizations aimed at generalizability, Lines 467–472.

3. More detail is needed on why certain datasets/years/resolutions were chosen and how results generalize.

**Response**

Thank you for your comment. Improving these detailed pieces of information is important for this study. We chose the Bijie landslide dataset mainly because it is one of the earliest and classic publicly released benchmarks for mountain landslide semantic segmentation, has expert carefully and independently annotated landslide ground truth, can support precise evaluation for typical scenes such as boundary ambiguity and scale heterogeneity, and is conducive to validating our method. We also selected Landslide4Sense2022 because its training subset comes from four typical events induced by different triggering factors and distributed across multiple regions (Iburi-Tobu, Hokkaido, Japan, 2018-09; Kodagu, Karnataka, India, 2018-08; Rasuwa, Bagmati Province, Nepal, 2015-04; western Taitung County, Taiwan, China, 2009-08), provides multimodal layers unified to 10 m (cloud-free post-event Sentinel-2, multi-temporal ALOS PALSAR DEM and slope, etc.), and the organizers withheld higher resolution location details to avoid external comparison; this design naturally emphasizes generalization evaluation across regions and across triggering factors. Compared with the “fine annotation for individual landslide objects” in Bijie, Landslide4Sense2022 is more engineering oriented and scene level in evaluation, where a single training sample often contains contiguous landslides and complex backgrounds, and can better test the robustness of methods under mid-resolution, multimodal, and class-imbalance conditions. Although we believe the current datasets can support model training, we have to admit that we do face difficulties in obtaining landslide inventories to build datasets, and at present the datasets that comply with CC BY 4.0 and provide both optical and DEM are only these two. We acknowledge that extreme cloud cover or other noise may still reduce confidence. In the future, we will further study the model performance after introducing such uncertainty to further optimize the model design.

**Changes made**

The authors added more details on the rationale for selecting specific datasets, Lines 275–282, and discussed future optimizations aimed at generalizability, Lines 467–472.

4. Current quality is not acceptable for publication; must be redrawn.

**Response**

Thank you for pointing this out. In the previous submission, the resolution was overly compressed in order to meet the file-size limitation. We have now readjusted the resolution to ensure that the figures both satisfy the quality requirements and remain within the upload size limit. Furthermore, in accordance with the editor’s suggestion and the journal’s requirements, we have replaced the unauthorized Google Maps image originally included in Fig. 4. As for Figs. 5 and 6, no copyright issues are involved, and both figures are appropriate for publication. The corresponding figure captions have been updated to explicitly clarify the copyright statement.

**Changes made**

The author re-uploaded all figures with higher quality and ensured the usability of intellectual property rights and the author revised the captions of the updated figures to ensure inclusion of copyright statements.

Third-party materials/permissions

Fig. 4

Source/Owner: Natural Earth (public domain).

License/Status: Public domain; no permission required; compatible with CC BY 4.0.

Credit line to include: “Basemap: Natural Earth II (Shaded Relief, Water, and Drainages), public domain. Made with Natural Earth.”

Fig. 5

Source/Owner: European Union/Copernicus; data provided by ESA.

License/Status: Copernicus Sentinel Data — free, full and open; governed by the Legal Notice on the use of Copernicus Sentinel Data and Service Information; no additional permission required.

Credit line to include: “Contains modified Copernicus Sentinel-2 data (2018), processed by the authors.”

Notes: No TripleSat (commercial) pixels are reproduced; only author-generated masks (trained/tested using the Bijie dataset) are displayed over Sentinel-2 imagery.

Fig. 6

Source/Owner: IARAI — *Landslide4Sense 2022 dataset*, DOI: 10.5281/zenodo.10463239.

License/Status: CC BY 4.0 (Zenodo record).

Credit line to include: “Landslide4Sense 2022 dataset (CC BY 4.0, DOI: 10.5281/zenodo.10463239).”

Figure caption

Fig. 4. Study area overview. Points of interest are shown over an open basemap. Basemap: Natural Earth II (Shaded Relief, Water, and Drainages), public domain. Made with Natural Earth. Figure © Authors, CC BY 4.0.

Fig. 5 Visual comparison on the Bijie dataset. Contains modified Copernicus Sentinel-2 data (2018), processed by the authors. No TripleSat pixels are reproduced; masks were trained/tested using the Bijie dataset. This replacement image is similar but not identical to the original and is for illustrative purposes only. Figure © Authors, CC BY 4.0.

Fig. 6 Visual comparison on the Landslide4Sense 2022 dataset. Panels use data from the Landslide4Sense 2022 dataset (CC BY 4.0, DOI: 10.5281/zenodo.10463239)

5. Needs a deeper scientific comparison, not only performance summary.

**Response**

Thank you for this suggestion. We conducted a more in depth and scientific comparative analysis and linked the changes in results and the observed differences to the modules.

**Changes made**

The authors standardized the expression in the results and analysis section and carried out a more in depth comparative analysis, see Lines 325–351, Section 3.3.3 Comparison with representative methods, Lines 375–377 and 383–387.

Response to Reviewer #1:

Abstract

(1) Lines 27: module should be deleted

**Response**

Thank you for pointing out this error; we have removed the redundant “moudle” at the corresponding location.

**Changes made**

The unnecessary term “moudle” was removed, see Line 31.

(2) Please add quantitative comparison to DEM fusion baselines in the Abstract, which can enhance the novelty of the manuscript.

**Response**

Thank you for the reviewer’s comment; this is helpful for enhancing the novelty of the manuscript.

**Changes made**

The authors added in the abstract, “We evaluate D2FLS-Net on the Bijie and Landslide4Sense 2022 datasets. On Bijie, the mean Intersection over Union (mIoU) reaches 88.77%, Recall 95.27%, and Precision 94.60%, exceeding the best competing model SegFormer by 7.96%, 7.90%, and 4.05%, respectively. On Landslide4Sense2022, mIoU 72.86%, Recall 82.55%, and Precision 93.30%, surpassing SegFormer by 7.06%, 6.56%, and 5.02%, respectively.” see Lines 37–41.

(3) The main novelty of the manuscript should be highlighted. Why do authors propose a novel to identify the boundary of landslides? The reasons for developing this study should be clarited.

**Response**

We are sorry that we did not clearly present our main innovations. The authors consider that effectively distinguishing landslide boundaries from background helps to achieve complete landslide recognition. This study aims to improve landslide recognition accuracy through the effective fusion of DEM and optical data. We rewrote the Introduction to clarify this point.

**Changes made**

We made major revisions and structural adjustments to the Introduction to highlight the addressed problem, see Lines 56–57 and 83–112.

(4) “remote sensing imaery” and “feature fusion” should be included in Keywords.
**Response**

Thank you for your suggestion; we have re-added it.

**Changes made**

Now the “Keywords: Landslide, Automatic recognition, Transformer, Remote sensing imagery, Feature fusion.”

Introduction/Methodology

(1) Lines 64-71,114-125. The authors should summary the main achievement of existing literature not simply listing study contents.

**Response**

Thank you for pointing this out, we have revised the description of this part and provided a clearer summary and our reflections on the related work.

**Changes made**

see Lines 71–74 ” Despite these studies respectively addressing class imbalance in landslide segmentation, reducing model complexity, and introducing attention mechanisms to improve landslide recognition accuracy, approaches based on convolutional neural networks face inherent limitations when applied to landslide segmentation.”

see line 95-96“These researchers used methods based on Transformers to effectively integrate global information and achieve landslide semantic segmentation. However, these studies remain confined to RGB three-band optical inputs.”

(2) Main contribution of the study should be described in Conclusion section, not the end of Introdution section.

**Response**

Thank you for your suggestion. We also, in line with your advice, reiterated and summarized our main contributions in the Conclusion section.

**Changes made**

We rewrote the final part of the Introduction, see Lines 114–127, and the Conclusion, see Section 5 Conclusion, Lines 475–494.

(3) Reorganize logic of Introduction section. The author should tell readers why developing this study.

**Response**

Thank you for pointing this out; in the revised version we made major revisions and structural adjustments to the Introduction to present a clearer logic.

**Changes made**

See Section 1 Introduction, Lines 49–127.

(4) Line 313 F1 equation is not found, and the F1 indicator is also not used to evaluate the performance of the model.

**Response**

Thank you for pointing out this issue. We confirm that we did not actually use the F1 metric in our study.

**Changes made**

Accordingly, we have removed the redundant content related to the F1 from the corresponding section of the manuscript.

Results and analysis

(1) The manuscript should elaborate on why two specific fusion stages (Swin-1 and Swin-4) were selected rather than intermediate stages. Provide ablation results testing alternative stage combinations.

**Response**

Thank you for your valuable suggestion. In our design, we deliberately incorporated the DEM-guided mechanism at the Swin-1 and Swin-4 stages, primarily for the following complementary reasons: (1) The Swin-1 stage has the highest spatial resolution (H/4), at which elevation-related discontinuities are most evident. Introducing DEM priors at this stage allows the shallow filters to be adjusted to focus more on terrain-salient pixels prior to RGB feature extraction. (2) The Swin-4 stage encodes the strongest semantic information with the widest receptive field. At this stage, the cross-attention mechanism enables the RGB queries to obtain DEM contextual information consistent with the global geomorphology, thereby alleviating the issue of modality inconsistency in later stages.

The intermediate stages, by contrast, neither provide the finest geometric details nor capture the richest semantic information, and thus their practical effectiveness is limited.

**Changes made**

To further validate this design, we have added an ablation study in which the two DEM-fusion stages were systematically interchanged across all stage combinations. Experimental results on both datasets consistently demonstrate that the (Swin-1, Swin-4) combination achieves the best performance in terms of mean Intersection over Union (mIoU), Recall, and Precision, confirming the rationality of the dual-stage design. See section 3.3.2 Ablation studies on the data fusion stages and Table4

(2) 700 samples are too few, which can not fully train deep learning models. From Table 1, the number of weighted coefficient (w), and bias (b) are over 1000. The authors should develop cross-validation strategy to illustrate the model robustness.

**Response**

We agree and have added a five-fold stratified cross-validation on both datasets (Bijie and Landslide4Sense 2022), creating folds at the image/patch level without leakage. Training protocol and hyper-parameters are kept identical across folds. We report ROC curves with mean ±1 SD bands and mean AUC ± SD for each model (new **Fig. 7**).

**Table 1 Cross-validation**

| **Method** | **Bijie** | **Landslide4Sense 2022** |
| --- | --- | --- |
| **D2FLS-Net** | **0.95 ± 0.01** | **0.91 ± 0.01** |
| **SAM（ViT-B）** | 0.89 ± 0.01 | 0.80 ± 0.01 |
| **SegFormer-B2** | 0.91 ± 0.01 | 0.82 ± 0.02 |
| **UNet** | 0.88 ± 0.02 | 0.79 ± 0.02 |
| **HRNet** | 0.90 ± 0.02 | 0.81 ± 0.02 |
| **ResNet50** | 0.84 ± 0.01 | 0.77 ± 0.02 |

The narrow-to-moderate bands observed in Fig. 7 indicate stable generalization under resampling. We also clarify that Table 1 enumerates backbone scaling settings rather than the total count of (w, b). Overfitting is mitigated by Adam with weight decay, data augmentation and the new cross-validation analysis.

**Changes made**

See section 3.3.4 Cross-validation – lines 435-448.

Conclusions:

(1) This section should tell readers what main contributions of the manuscript is.

**Response**

Thank you for your suggestion; we rewrote the Conclusion section.

**Changes made**

please see Line 475-494。

Figures

(1) The quality of the figures is too poor to recognize texts.

**Response:**We appreciate your comment regarding the figure quality. In fact, we do have high-resolution, high-quality versions of these figures. However, due to file size limitations for uploads on the submission system, we had to reduce their image quality during the initial submission.

**Changes made**

To address this issue, we have now re-uploaded the high-resolution figures for your reference.

Response to Reviewer #2:

- this research was conducted in Bijine City and is a case study. Why isn’t this mentioned in the title?

**Response：**We appreciate the concern about geographic scope. Our work is not a single‐site case study centered only on Bijie. The manuscript evaluates the proposed D2FLS-Net on two public benchmarks with distinct sensing characteristics and geographies: (i) the Bijie dataset (0.8 m RGB + DEM) from Guizhou, China, and (ii) Landslide4Sense 2022, a multi-sensor benchmark compiled from multiple regions across different countries. The second dataset was included precisely to test generalization across resolutions, sensors, and terrains, and our cross-dataset experiments (Tables 4–5; Figs. 5-6) and 5-fold cross-validation (Figs. 7) show consistent improvements, indicating that the method is not tailored to a single locale.

Because the primary contribution is methodological—a dual-stage DEM-guided fusion transformer and a terrain-aware pixel-adaptive context module—the title intentionally emphasizes the approach rather than a particular study area. We believe this aligns with the paper’s scope and avoids implying that the method is region-specific. The geographic provenance and characteristics of both datasets are clearly stated in the “Data description” and “Experimental setup” sections, together with quantitative and qualitative comparisons across the two benchmarks.

- in the abstract, you proposed the “Dual-Stage DEM-guided…” method before presenting the results. It’s possible that after reviewing the results, you might decide not to propose this method. Please either correct this or remove it from the abstract section.

**Response**

Thank you for your suggestion; stating “we propose Dual-Stage DEM-guided …” may be insufficiently rigorous.

**Changes made**

We corrected this part to “To address this, We propose a Swin-Transformer–based framework, Dual-Stage DEM-guided Fusion Transformer for landslide segmentation (D2FLS-Net), which embeds terrain features via two modules” see line 29.

- using the acronym “D2FLS-Net” in the title without sufficient explanation might be ambiguous for some readers. It’s better to use the full equivalent of this term in the title, at least.

**Response**

The authors are sorry about this issue. A lack of understanding of the journal requirements for “Full Title” and “Short Title” led to our failure to set the correct title in the submission system; we apologize for the misunderstanding. “D” denotes DEM guided, and “2” denotes Dual Stage.

**Changes made**

Our full title is D2FLS-Net:Dual-Stage DEM-guided Fusion Transformer for landslide segmentation. The authors have updated the title information in the system.

- in line 6 of the abstract, the word “module” has a typo that needs to be corrected.

**Response**

First and foremost, we apologize for this oversight. As non-native English speakers, we confused the commonly used software package names with the term "module," which led to the typo.

**Changes made**

To ensure no similar issues remain, we have thoroughly rechecked all relevant spellings throughout the manuscript and made the necessary corrections.

- in abstract: Given the claim of “Robust” in the title, the abstract should have elaborated more on how this robustness is achieved (e.g., does the model perform consistently under various data conditions, vegetation cover, or landslide types?). Although the mention of improved Recall in hazy and vegetated areas hints at it, it could have been highlighted more prominently.

**Response**

We highly appreciate the reviewer’s opinion. The authors are sorry for the misunderstanding caused by technical issues in our translation. Although this may sound like evading responsibility, a lack of understanding of the journal requirements for “Full Title” and “Short Title” led to our failure to set the correct title in the submission system. The authors did not intend to emphasize “robustness” but rather to convey that the model is optimized based on the two proposed modules. Although two types of landslides can be seen clearly from the dataset snapshots, namely blurred and vegetation covered landslides and landslides with obvious visual differences from the background, the dataset does not label specific landslide types or state the time difference between the landslide occurrence and the data acquisition. For rigorous expression, we therefore do not emphasize new landslides and old landslides, and we do not highlight that the model specifically targets any particular landslide type.

**Changes made**

In any case, we reset and corrected the title in the submission system. Our full title is D2FLS-Net: Dual-Stage DEM-guided Fusion Transformer for landslide segmentation.

- In line 3 of the abstract: Does this imply that previous models didn’t benefit from DEM in some scenarios, and this model always does? More clarity is needed.

**Response**

Yes, our model ensures that DEM provides a stable gain for landslide recognition. Previous approaches based on simple channel level concatenation or single stage fusion make it difficult for DEM to maintain a stable gain across different landslide types. When only channel level concatenation at the input (from 3+1 to 4 channels) is used, it usually yields an effective gain for “old” landslides, in which vegetation cover and weathering or erosion make the visual difference between the landslide body and the surrounding background inconspicuous and the boundaries blurred; however, for “new” landslides, in which the landslide body and the surrounding background show clear visual differences, for example the body is bare soil or gravel while the background is vegetation cover, the landslide and its boundaries can already be well recognized from RGB imagery, and simply adding DEM introduces redundancy, interferes with the original precise segmentation, and degrades performance. When fusion is applied only at high level semantic features, the effect on landslides with clear visual differences is limited, but for landslides with blurred boundaries and small background differences, the DEM features become abstracted and sparse, leading to information loss.

**Changes made**

The authors revised the abstract to more clearly explain this part. ”Although Transformers surpass convolutional neural networks in modeling long-range contextual dependencies, channel-level or feature-level fusion strategies provide only intermittent terrain cues, leading models to underutilize digital elevation model (DEM) information and to lack fine-grained adaptability to terrain variability. ”

- In the keywords section: You could also use “DSDF,” “T-PACE,” “Fusion,” and “DEM-Guided” to increase the se archability of the article.

**Response**

Thank you for the suggestion. We have added "Feature Fusion" to the keywords section.

**Changes made**

Now the “Keywords: Landslide, Automatic recognition, Transformer, Remote sensing imagery, Feature fusion.”

- Various types of landslides exist, and their causal factors differ in some cases. This point must be taken into consideration.

**Response:**Yes, your review is entirely correct. This is consistent with the core idea we intended to convey in our paper—that for a semantic segmentation task such as landslide recognition, improvements should not only be considered from the perspective of deep learning or optical imagery, but also by taking into account the characteristics of the target objects and integrating the concept of landslide susceptibility mechanisms into the model design process.

This is also exactly what we plan to pursue in future work. For example, regardless of vegetation coverage, incorporating InSAR data on creeping-type landslides during the incubation stage may be more effective than using DEM; for rainfall-induced landslides, integrating annual rainfall data would be more appropriate; and for earthquake-triggered coseismic landslides, incorporating peak ground acceleration (PGA) would be preferable. However, in the present study: (1) our approach remains targeted rather than comprehensive; (2) since we used a publicly available landslide dataset, even from the dataset description and related publications, the providers did not release detailed information or explicit labeling of landslide types; and (3) although there are many different types of landslides with diverse triggering factors, previous susceptibility evaluation studies consistently show that DEM, as a representation of geomorphology, ranks among the most important features regardless of landslide type. This is why we have adhered to the fusion of DEM with optical remote sensing imagery.

In any case, we sincerely thank you for this insightful suggestion, which we believe will be very helpful in guiding our future research.

- L. 52: is it truly 100% feasible in reality to use highly accurate automated systems for identifying these features?

**Response:** That’s really a good question, it is a challenging problem to use highly accurate automated systems in reality. However, we have made substantial progress, and with the development of subsequent algorithms and the expansion of relevant datasets, drawing on the successful applications of deep learning in other fields, we can believe that the performance can reach a practically usable level.

- L. 62: after introducing the challenges and mentioning CNNs, there is a need for a clearer paragraph explaining why existing CNN methods (or even general transformers) are insufficient for these challenges and why the proposed approach (D2FLS-Net) is necessary. This knowledge gap should be articulated more explicitly to provide a stronger justification for the innovation.

**Response**

Thank you for your suggestion. In the revised version, we have added an additional paragraph to reasonably explain why the existing CNN and general conventional transformer methods do not perform well enough.

**Changes made**

Please See lines 71-82 “Pixels at different positions within a landslide body require different levels of contrast with the background, and the fixed receptive fields of CNNs often struggle to balance broad context modeling with precise boundary delineation.”

and lines 109-115 “However, Li et al. found that simple early fusion like stacking DEM as an additional channel often fails to fully exploit topographic cues and may even introduce noise, which can cause the Hughes phenomenon and reduce the recognition performance for landslides whose bodies show clear visual differences from the surrounding background. Some more advanced schemes adopt late fusion or parallel fusion, but during feature extraction these approaches can lead to loss of DEM information, thereby limiting the model’s discriminative capability in complex terrain where the visual differences between landslide bodies and background are small.”

- L 64: the reference to “Yang et al.” without sufficient explanation of their achievement is a bit abrupt. It would have been better to briefly explain what their work was and what limitations it had that this paper intends to address.

**Response**

Thank you for your suggestion; we rewrote the Introduction. In addition to the overarching statement “Convolutional neural networks (CNNs), in particular, have achieved success in various remote sensing segmentation tasks and have been widely explored for landslide mapping” to introduce the work of Yang et al., we also summarized their work so as to lead to the limitations of their work and the reason for turning to the Transformer.

**Changes made**

See lines 62-65 ” Convolutional neural networks (CNNs), in particular, have achieved success in various remote-sensing segmentation tasks and have been widely explored for landslide mapping. Yang et al. compared U-Net, DeepLabv3+ and PSPNet to identify optimal configurations for landslide detection.”

- in the introduction: the introduction of the article should include a section on the general overview of the research and another section on previous research. These sections should be clearly distinguishable from each other. In this article, this is not observed, and in some sections (e.g., L. 90), the reader becomes confused about which part of the introduction they are in.

**Response**

Sorry for confusing you. We have revised the related description in our latest manuscript **Changes made**

Regarding the prior research section, please see Lines 58–113. Regarding the overall overview of the study, please see Lines 114–127.

- L. 125: Although the abstract referred to “inconsistent benefits of combining DEM with optical data,” the introduction has not yet addressed this issue and its reasons (e.g., why previous models struggled to combine these data). This topic requires fundamental justification.

**Response**

Thanks for your constructive advice. In the revised Introduction the authors rewrote and supplemented this issue and its causes.

**Changes made**

Please see lines 109-115 “Li et al. found that simple early fusion like stacking DEM as an additional channel often fails to fully exploit topographic cues and may even introduce noise, which can cause the Hughes phenomenon and reduce the recognition performance for landslides whose bodies show clear visual differences from the surrounding background. Some more advanced schemes adopt late fusion or parallel fusion, but during feature extraction these approaches can lead to loss of DEM information, thereby limiting the model’s discriminative capability in complex terrain where the visual differences between landslide bodies and background are small.”

- L. 137: although the DSDF and T-PACE modules seem innovative, it must be clearly shown how these modules not only improve performance but also specifically solve particular challenges (such as boundary ambiguity and inconsistent DEM benefits). This requires providing a deeper analysis.

**Response**

We highly appreciate the reviewer’s opinion and have made corresponding revisions accordingly.

**Changes made**

At the end of the Introduction we emphasized the addressed problem and how the modules resolve it, please see Lines 109–129. Meanwhile, in the Results and Analysis section we conducted an in depth analysis linking the model’s performance improvements to the roles of the modules, please see Section 3.3.

- given the claim of “Robust” in the title, the paper should include more analyses demonstrating how the model performs against variations in data quality, landslide types, and different environmental conditions (e.g., in different seasons or with varying vegetation cover). Have experiments been conducted on noisy or diverse data?

**Response**

We highly appreciate the reviewer’s opinion. The authors are sorry for the misunderstanding caused by technical issues in our translation. Although this may sound like evading responsibility, a lack of understanding of the journal requirements for “Full Title” and “Short Title” led to our failure to set the correct title in the submission system. The authors did not intend to emphasize “robustness” but rather to convey that the model is optimized based on the two proposed modules. Although two types of landslides can be seen clearly from the dataset snapshots, namely blurred and vegetation covered landslides and landslides with obvious visual differences from the background, the dataset does not label specific landslide types or state the time difference between the landslide occurrence and the data acquisition. For rigorous expression, we therefore do not emphasize new landslides and old landslides, and we do not highlight that the model specifically targets any particular landslide type.

**Changes made**

In any case, we reset and corrected the title in the submission system. Our full title is D2FLS-Net: Dual-Stage DEM-guided Fusion Transformer for landslide segmentation.

- L. 148: the details of the “baselines” and the rationale for their selection should be carefully explained. Does the comparison include methods specifically focused on combining DEM and optical data?

**Response**

Thank you for your valuable suggestion. We have provided an explanation of the details of the “baselines” and the rationale for their selection. These baselines were chosen as widely adopted and well documented representatives of the two main paradigms in geospatial semantic segmentation, namely Transformer based methods (SAM, SegFormer) and CNN based methods (U-Net, HRNet, ResNet-50). They are widely used in remote sensing segmentation research, cover complementary architectural designs (foundation models that can facilitate segmentation, hierarchical Transformer encoders, encoder decoder CNNs, and high resolution CNNs), and offer reproducible, community recognized reference points for fair comparisons under standard training protocols.

Unfortunately, our comparative analysis did not include methods that specifically focus on the fusion of DEM and optical data. The reason is that such specialized fusion methods, especially those for landslide related tasks, are often tailored to specific regions, or their models are not open source. These issues make it currently impossible or very difficult for us to obtain these methods.

Nevertheless, your suggestion remains highly valuable. The datasets we selected are also very representative, and many studies are based on these two datasets. Therefore, in the Discussion section we added a comparison between the proposed network and existing research results. This subsection clearly shows that on both datasets, namely the Bijie dataset and the Landslide4Sense 2022 dataset, our corresponding results exceed those of other methods that specifically focus on the fusion of DEM and optical data.

**Changes made**

Baseline details, please see Lines 128–129: “On the Bijie Landslides and Landslide4Sense2022 datasets, our model achieves superior landslide recognition performance compared with SAM, SegFormer, U-Net, HRNet, and ResNet-50.” The comparison with methods dedicated to the fusion of digital elevation model (DEM) and optical data is given in Lines 458–467.

- L. 148: although transformer models are powerful, their interpretability can be challenging. Do the authors have a plan to show how the model uses DEM and RGB information (e.g., with saliency maps or other interpretability techniques)?

**Response**

We agree that interpretability is important, particularly for risk-critical applications. The present study is scoped to the methodological question of how to fuse DEM with RGB to improve landslide delineation; therefore we prioritized fusion design and quantitative validation. In the revised manuscript, we acknowledge this limitation and outline concrete interpretability plans in discussion

**Changes made**

See lines 468-475 “The present work has several boundaries. The method attains strong accuracy related metrics, but its interpretability may be challenging. Although it is designed on a transformer to learn more complex landslide features, it still requires more global landslide data for training, and it should be appropriately lightened to fit deployment in automated landslide recognition systems. Therefore, we consider the most useful next steps to be optimization of time complexity, construction of more suitable datasets for training and testing to improve practicality and robustness, and provision of interpretability through cross attention visualization and stage wise saliency to support the method.”

+ how does D2FLS-Net address these problems with “dual-stage topographic injection,” “cross-attention,” and “context enhancement”? These should be introduced as direct solutions to the problems raised in the introduction.

**Response**

Thank you for your comments; we rewrote and refined the Introduction and the Methods section to ensure correspondence between the problem and the solution.

**Changes made**

Please see lines 109-115, ”However, Li et al. [28] found that simple early fusion like stacking DEM as an additional channel often fails to fully exploit topographic cues and may even introduce noise, which can cause the Hughes phenomenon and reduce the recognition performance for landslides whose bodies show clear visual differences from the surrounding background. Some more advanced schemes adopt late fusion or parallel fusion [29], but during feature extraction these approaches can lead to loss of DEM information, thereby limiting the model’s discriminative capability in complex terrain where the visual differences between landslide bodies and background are small.”

189-192, “To address the problem that fusing DEM features at the low level channel stage or at the high level semantic stage causes landslides with different manifestation forms to be unable to simultaneously and stably benefit from DEM derived topographic cues.”

199-202, “Early DEM guidance does not fully fuse DEM in a way that would disturb landslides with clear visual differences. Instead, it acts in a weighted form to assist the optical data in the semantic segmentation of landslides. This multiplicative gain retains radiometric cues while emphasizing terrain-salient pixels, improving downstream feature extraction for low-contrast landslides.”

216-220, “High level cross attention fuses DEM and optical data once again at the high level semantic feature stage and enables interaction between the two features to address the missed detection problems for landslides with blurred boundaries or vegetation occlusion. At this point, the DEM features have been sparsely extracted and abstracted to a certain level, and likewise will not interfere with the recognition of landslides that show clear visual differences.”

+ the abstract mentions “inconsistent benefits of fusing DEMs,” but the introduction has not yet fully clarified this problem for the reader. It should be explained why simply “adding DEM” to CNNs or transformers does not always yield optimal results. Is this due to incorrect alignment of visual and topographic features? Or due to differences in the nature of their information?

**Response**

We would like to answer from two perspectives why simply adding a digital elevation model (DEM) to convolutional neural networks (CNNs) or Transformer models does not always yield optimal results. For so called “new landslides” where the landslide body is clearly distinguishable from the surrounding background, optical data alone can already recognize landslides well; in this case, fully injecting DEM may cause the Hughes phenomenon, meaning that once the feature dimensionality passes a critical point, further increases can degrade the performance of the classifier. For landslides whose bodies are not clearly distinguishable from the surrounding background and whose boundaries are not visually obvious, regardless of the specific cause or the time elapsed since the event, for example vegetation regrowth on landslides in formerly vegetated areas or long term weathering and erosion in non vegetated areas that make the landslide body almost indistinguishable from the background, a single fusion scheme of DEM and RGB, whether only feature level fusion or only high level semantic fusion, may fail to provide stable gains because it can introduce redundancy or lead to the loss of DEM cues. Therefore, simply adding DEM to CNNs or Transformer models does not always yield optimal results.

**Changes made**

We rewrote the Introduction and fully clarified the issue of the inconsistency in the benefits of fusing digital elevation models (DEMs). Please see Lines 109–115. “However, Li et al. [28] found that simple early fusion like stacking DEM as an additional channel often fails to fully exploit topographic cues and may even introduce noise, which can cause the Hughes phenomenon and reduce the recognition performance for landslides whose bodies show clear visual differences from the surrounding background. Some more advanced schemes adopt late fusion or parallel fusion [29], but during feature extraction these approaches can lead to loss of DEM information, thereby limiting the model’s discriminative capability in complex terrain where the visual differences between landslide bodies and background are small.”

+ why are CNNs, despite their powerful capabilities, still insufficient, paving the way for the Transformer?

**Response**

The strength of CNNs lies in the inductive bias of local perception and translation equivariance: small kernels extract edges and textures within neighborhoods, and stacked layers gradually enlarge the receptive field, yielding strong performance on stable local patterns. However, this fixed and local modeling brings three structural limitations. First, long range dependency capture is inefficient, since linking distant regions requires multiple downsampling and stacking, which dilutes contextual information through pooling. Second, relation modeling is constrained, because convolution shares the same kernel across all positions and thus struggles to adapt weights and dependency structure to the content. Third, generalization across scale and deformation often relies on hand crafted modules such as pyramids, atrous designs, or FPN, which increase complexity while still risking the omission of global cues across scales and regions. Transformers use self attention to directly connect any two positions, providing a naturally global and content dependent receptive field that more easily builds long range dependencies and global consistency. Their attention weights can adapt between detail and semantics with less restriction from fixed kernel sizes. Through cross attention they also realize multimodal alignment in a natural way, such as between imagery and topography. Coupled with large scale pretraining and high parallelism, Transformers offer a more general solution and a higher performance ceiling for the inherent limitations of convolution in global relations, modeling across scales, and multimodal fusion.

**Changes made**

Please see lines 76-82. “Pixels at different positions within a landslide body require different levels of contrast with the background, and the fixed receptive fields of CNNs often struggle to balance broad context modeling with precise boundary delineation. For example, in mountainous areas with vegetation cover, a landslide body that appears similar to bare soil shows a clear visual contrast with the vegetated background. When the receptive field is too small, critical background information may be missed, which is unfavorable for determining that the pixel at that location belongs to a landslide, when the receptive field is too large, fine details may be overlooked, preventing a precise depiction of the landslide boundary.”

- L. 171: Why only these resolutions? Is there a specific reason?

**Response**

These resolutions and channel widths follow the canonical hierarchical design of Swin Transformer. We did not intentionally modify them, as this design is well-balanced for our tasks.

- L. 176: Typo!

**Response**

Thank you for pointing out the typo. We have revised the corresponding part.

- from lines 178 to 199: None of the equations are referred to in the text of the article, nor do they have any references.

**Response**

Thank you for pointing out this issue; we have re-cited the corresponding equation in the main text using the correct citation format.

**Changes made**

Please see lines 165-266

- L. 200: Typo!

**Response**

Thank you for pointing out the typo. We have revised the corresponding part.

- L. 256: Generally, In which part of the article did you consider this?

**Response**

In general, forest vegetation can, through root reinforcement, interception of rainfall, and promotion of infiltration, significantly reduce the occurrence of shallow sliding; however, in regions strongly driven by tectonic and climatic factors, the presence of forest does not imply the absence of landslides. Taking Bijie in Guizhou and the southwestern flank of the Tibetan Plateau as examples, the former lies at the northern margin of the karst plateau where fault activity and extreme rainfall coexist, the rock and soil mass is fragmented, and local slope cutting for road construction is superimposed, which limits the slope stabilization effect of coniferous or fast growing forests on steep slopes; the latter is located in the Hengduan Mountains and western Sichuan seismic corridor, where frequent strong earthquakes, such as the Luding earthquake, can trigger tens of thousands of coseismic landslides even in areas with high forest cover, indicating that strong triggers can override the stabilizing effect provided by vegetation. The four study areas in the Landslide4Sense 2022 dataset are mostly mountainous forest regions and are dominated by strong triggers, namely Iburi in Hokkaido, Japan in 2018 with an earthquake compounded by typhoon rainfall that induced large scale shallow landslides, Kodagu in Karnataka, India in 2018 with monsoon rainstorms along the Western Ghats that caused many landslides, Gorkha in Nepal in 2015 with an Mw7.8 earthquake, and Taiwan in 2009 with extreme rainfall from Typhoon Morakot. These regions share common features, including dense faulting, frequent earthquakes and intense rainfall events, highly fragmented slope bodies and cover layers, and superimposed human disturbances such as landscape fragmentation and slope cutting for roads, which together lead to landslides remaining prone even where a considerable proportion of forest cover exists.

**Changes made**

Please see lines 97-101 “For regions such as southwestern China, Nepal, and Japan, although the frequency of landslides in forested areas is considered generally lower, these areas lie near active fault zones and experience extreme rainfall and other climatic drivers, coupled with ecological fragmentation, landslides still occur frequently despite vegetation cover.”

- from lines 263 to 283: None of the equations are referred to in the text of the article, nor do they have any references.

**Response**

Thank you for pointing out this issue. We have revised the relevant content you noted accordingly—specifically, we have now referenced the equations in the main text and supplemented the necessary references for them.

**Changes made**

Please see lines 165-266

- L. 293: Why these specific months and year? Given that there’s about a 7-year time gap to the present, is it logical to use images from 2018?

**Response**

First, this study focuses on landslide recognition after the event rather than landslide process modeling, so it does not involve inference of dynamic mechanisms before and after the trigger; the samples are used only for posterior segmentation learning, and the time gap does not constitute a “temporal mismatch” for semantic segmentation training. If “temporal generalization” is discussed, the Landslide4Sense 2022 dataset itself is a benchmark constructed across regions and across time phases, covering four areas with data spanning 2015–2021, and is used to assess model robustness under spatiotemporal transfer [1-2].

From the perspective of data availability and comparability, the authors acknowledge that Bijie and Landslide4Sense 2022 are not the “latest” relative to 2025, but they are at present two authoritative landslide segmentation resources that publicly provide both RGB and the corresponding DEM and terrain derived layers, and they have been widely adopted by subsequent studies. For example, on Landslide4Sense 2022, UNet pyramid, GMNet, and lightweight segmentation methods have all reported systematic experiments [3-5]. On the Bijie dataset, the official release explicitly includes RGB and DEM and has been used by many works to validate the conclusion that RGB plus DEM is superior to RGB alone and to design multimodal structures [6-8]. There are also studies that conduct comparative evaluation on both Bijie and Landslide4Sense 2022 to examine cross domain generalization performance [9]. Accordingly, we chose these two datasets as the main experimental platforms to ensure the availability of topographic priors, that is DEM, and reproducible experiments, while also meeting the need for evaluation across regions and across time phases.

[1] Fang C, et al. A globally distributed dataset of coseismic landslide mapping via multi-source high-resolution remote sensing images (GDCLD). Earth System Science Data. 2024;16:4817–4842. doi:10.5194/essd-16-4817-2024.
[2] Ghorbanzadeh O, Xu Y, Ghamisi P, et al. Landslide4Sense: Reference benchmark data and deep learning models for landslide detection. IEEE Transactions on Geoscience and Remote Sensing. 2022;60:15209.
[3] Kaushal A, Gupta AK, Sehgal VK. A semantic segmentation framework with UNet-pyramid for landslide prediction using remote sensing data. Scientific Reports. 2024;14:30071. doi:10.1038/s41598-024-79266-6.
[4] Wang K, et al. A novel network (GMNet) for semantic segmentation of landslides. Applied Soft Computing. 2024;152:111007.
[5] Dai J, et al. Using lightweight method to detect landslide from satellite images. Engineering Applications of Artificial Intelligence. 2024;(online ahead of print).
[6] WHU-GPCV. Bijie Landslide Dataset.
[7] Qin S, et al. Landslide detection from open satellite imagery using distant domain transfer learning. Remote Sensing. 2021;13:3383. doi:10.3390/rs13173383.
[8] Zhou N, et al. A multiscale attention segment network-based semantic segmentation method for landslide detection. Remote Sensing. 2024;16:1712. doi:10.3390/rs16101712.
[9] Jia L, et al. Recognizing landslides in remote sensing images based on deep learning. GIScience & Remote Sensing. 2024;(online ahead of print).

- from lines 308 to 313: None of the equations are referred to in the text of the article, nor do they have any references.

**Response**

Thank you for pointing out this issue. We have revised the relevant content you noted accordingly—specifically, we have now referenced the equations in the main text and supplemented the necessary references for them.

**Changes made**

Please see lines 313-317

- L. 306: FN, TN, FP, TP must be clearly defined as to what factors they represent and how they are calculated.

**Response**

Thank you for the suggestion. We have revised the Evaluation Metrics paragraph to explicitly define TP/FP/TN/FN at the pixel level, state how predictions are obtained, and clarify how we compute IoU, Precision, Recall, and mIoU.

**Changes made**

Please see lines 303-311

- L. 326: the results section should preferably start with a general introduction and text rather than a table. This point must be taken into consideration.

**Response**

We agree. We have added a short overview paragraph at the beginning of table1

**Changes made**

Please see lines 330-333

- in the discussion and conclusion sections: this section is written very generally and briefly, lacking any relevant discussion and merely reviewing the results from the previous section. Furthermore, in this section, you have not compared your research findings with those of other researchers. This section definitely needs to be rewritten. Also, please provide some relevant suggestions related to your research, especially concerning the study area, that would lead to solving a problem (please pay special attention to this point).

**Response**

hanks for such constructive advice, we rewrite the discussion and conclusion sections

**Changes made**

Please see lines 461-506

- page 36: The quality of the figures is very low.

**Response:** We appreciate your comment regarding the figure quality. In fact, we do have high-resolution, high-quality versions of these figures. However, due to file size limitations for uploads on the submission system, we had to reduce their image quality during the initial submission.

**Changes made**

To address this issue, we have now re-uploaded the high-resolution figures for your reference.

- page 39: No cartographically standard map of the study area has been provided. Unfortunately.

**Response**

Thank you for your comment. We have re-provided the standard maps for all regions pertaining to the dataset.

**Changes made**

Fig. 4 Study area overview. (Points of interest are shown over an open basemap. Basemap: Natural Earth II, public domain. Made with Natural Earth. Figure © Authors, CC BY 4.0.)

- page 40: It appears that SAM identifies areas with more severe landslides much more accurately!

**Response**

When compared against the standard labels, our proposed model achieves better landslide recognition performance across all datasets—this superiority is evident both in terms of visualization results and quantitative comparisons.

Fig. 5 Visual comparison on the Bijie dataset. Contains modified Copernicus Sentinel-2 data (2018), processed by the authors. No TripleSat pixels are reproduced; masks were trained/tested using the Bijie dataset. This replacement image is similar but not identical to the original and is for illustrative purposes only. Figure © Authors, CC BY 4.0.

Fig. 6 Visual comparison on the Landslide4Sense2022 dataset.(CC BY 4.0, DOI: 10.5281/zenodo.10463239)

+ were any field visits conducted to the landslides extracted from the images to verify the accuracy of the image-derived results?

**Response**

First, it must be emphasized that we used publicly available datasets, which provide only optical imagery and DEM data but do not include specific landslide coordinate information. Therefore, field investigation is indeed not feasible. This is, admittedly, a limitation of our study. Nevertheless, the datasets do provide ground-truth labels, as illustrated in the labels shown in Figures 5 and 6.

+ none of the figures have legends! Unfortunately!

**Response**

Thank you for pointing out this oversight.

**Changes made**

We have added legends to the visual comparison figures to clearly indicate the landslide regions and background areas.

+ the current paper is theoretically very strong but has fundamental weaknesses in its practical and implementable aspects.

**Response**

Thank you for highlighting the practicality concerns. We have substantially strengthened the implementable aspects by (1) providing code as required, (2) providing the experimental settings, (3) reporting inference time, and (4) conducting ablation studies. These updates increase the practicality of our work to some extent. Although we are currently facing difficulties in research funding, in future work we will continue to refine the practical aspects and promote the implementation of such methods in real world scenarios.

**Changes made**

Please see Section 3.3.2 Ablation studies on the data fusion stages, Lines 437–446, and Section 3.3.4 Cross validation.

+ the paper does not have a good literature review and has not adequately answered the question of why this research was conducted.

**Response**

Sorry for lacking sufficient descriptions of related works and motivation, we have supplemented these in our introduction

**Changes made**

Please see the Introduction.

+ based on the article, it can be predicted that the paper was prepared quickly, and not much time was spent on improving its quality and quantity.

**Response**

As a novice facing an imminent graduation deadline, I sincerely apologize for the various errors caused by my oversight. I am grateful for all of your corrections and comments. We have carefully revised the manuscript in accordance with the reviewers’ suggestions, and we look forward to receiving your further feedback on this revision.

+ what are the CELL SIZES of the DEMs you used?

**Response**

Thank you for your question. For the Bijie dataset, the DEM cell size is 0.8 meters.

For the Landslide4Sense 2022 dataset, the DEM cell size is 10 meters.

+ does the time of landslide occurrence also affect your research results? Meaning, for example, a landslide that occurred in 2014 versus one in 2018. How can the impact of this 4-year difference be eliminated so as not to affect the results?

**Response**

First, we emphasize that we consider the time of landslide occurrence not to affect our results. This study targets the appearance of landslides as recorded in the final imagery (i.e., it recognizes landslides after they have occurred). The paper is not a change-detection study and does not involve multi-temporal analysis or the landslide process. Regardless of when a landslide occurred, only the image acquisition time determines the training samples we ultimately use. A landslide that occurred in 2014 with training samples produced from 2014 imagery and a landslide that occurred in 2018 with training samples produced from 2018 imagery are, in essence, both “new landslides” and do not differ. If only 2018 imagery is used to create training samples for landslides that occurred in both 2014 and 2018, then for the 2014 landslide the sample produced from 2018 imagery will obviously differ visually from the sample produced from 2014 imagery. This is precisely the key problem addressed in our paper.

The purpose of our model is to recognize landslides after they have occurred which, under various influences (e.g., re-vegetation of the landslide body, weathering and erosion), exhibit a temporal difference relative to newly formed landslides; during this period, the visual contrast between the landslide body and the surrounding background diminishes, making it necessary to consider terrain features rather than relying solely on optical features. If the landslide happened to be reactivated during those four years, it would again become a “new landslide.”

If insists on calling this “eliminating differences,” it is achieved by effectively fusing DEM data (ensuring that recognition of landslides with blurred boundaries is improved without degrading recognition of landslides with clear boundaries), rather than relying only on optical data.

At the same time, for the two datasets used, the original Bijie dataset description only emphasizes that the imagery was acquired in 2018, while the Landslide4Sense2022 release notes only emphasize the region. Therefore, the times of landslide occurrence are unknown; nevertheless, it is evident that Bijie is a mountainous, vegetated area and, judging from the sample snapshots, contains landslides with diverse appearances.

+ can the volume of material displaced by the landslide and the area of the landslide also affect the results?

**Response**

It affects the results and, as stated in the main text, constitutes a challenge. Although the volume of displaced material in a landslide cannot typically be directly obtained from two-dimensional imagery, it exhibits a stable power-law relationship with area. From the perspective of landslide area, the authors consider that if a landslide is smaller than the pixel size, this is no longer a problem that can be solved by method design; data quality determines the final result, and improvements in method/model design merely aim to approximate this result. Moreover, when a landslide is below a certain scale, its hazard decreases. Although this study does not focus on the identification of small landslides, the small-landslide test sample (b) in Figure 6 was still recognized owing to the class-imbalance loss design, and we enlarged it in the lower-right corner.

+ does the orientation of the hillslopes also affect the results?

**Response**

Terrain features such as slope and aspect are helpful for landslide recognition; however, they are already encompassed within the DEM. Although other studies have explicitly extracted slope and aspect, or have included an extraction step when designing DEM–optical imagery fusion, excessive inter-feature correlation can lead to overfitting in linear settings, which must be taken into account. We view this as a promising future direction rather than a limitation of the current findings.

+ does the general shape of the hillslopes also affect the results?

**Response**

Yes—hillslope shape (e.g., concave/convex planform and profile curvature, ridge/valley position, local relief) can influence segmentation performance. However, as with slope and aspect, these variables are preserved as indirect information within the DEM. Accordingly, our approach fuses DEM data with optical imagery, rather than fusing slope with RGB optical imagery or hillslope shape with RGB optical imagery. We view this as a promising future direction rather than a limitation of the current findings.

+ does the actual shape of the hillslopes (e.g., convexity, concavity, divergence, convergence, etc.) also affect the research results?

**Response**

Answer to the previous question. In principle, hillslope morphology influences how landslide scars appear in topography and optics. We use this appearance to identify landslides. In our study—which targets post-event segmentation rather than susceptibility or hazard. We view this as a promising future direction rather than a limitation of the current findings.

+ to improve the logical flow, the introduction should include a clear “Our Contributions” section that summarizes the innovations of D2FLS-Net in bullet points, with reference to the challenges raised. This helps the reader know exactly what to expect from the rest of the paper.

**Response**

Thanks for your advice, and we have implemented this in the line 118-129.

**Changes made**

(1) We construct a Dual-Stage DEM-Guided Fusion (DSDF) module, which in the early stage guides and assists optical semantic segmentation for landslide recognition in the form of weights, and in the later stage enables interaction with optical features at the high level semantic feature stage, ultimately achieving effective fusion of optical data and DEM data, overcoming the limitations of one time fusion and ensuring persistent topographic guidance throughout the process from low level edge features to high level semantic features.

(2) We construct a Terrain-aware Pixel-wise Adaptive Context Enhancement (T-PACE) module, which addresses the limited receptive-field range of dilated (atrous) convolutions; through DEM-gated multi-dilation convolutions, the model is endowed with dynamic, location-specific receptive fields, enabling fine boundary preservation.

(3) On the Bijie Landslides and Landslide4Sense2022 datasets, our model achieves superior landslide recognition performance compared with SAM, SegFormer, U-Net, HRNet, and ResNet-50.

+ how confident are you in the results of the current research such that if you were asked to implement it as a pilot project in one area, and if successful, to implement the results across an entire province, country, or even globally; would you agree to this proposal?

**Response**

We are encouraged by the promising results of the current research and have confidence in its potential for practical application. At the same time, we recognize that real-world implementation may involve additional challenges that require further testing and refinement. We would therefore welcome the opportunity to begin with a pilot project in a specific area, and, if successful, we believe the approach could be gradually scaled up to larger regions, including provincial, national, or even global levels.
