# Supplementary material for: D2FLS-Net: Dual-Stage DEM-guided Fusion Transformer for landslide segmentation
Source: PLoS One. 2025 Nov 26;20(11):e0337412. doi: 10.1371/journal.pone.0337412 (PMC12654934; doi:10.1371/journal.pone.0337412)
Supplement: Attachment — Submitted filename: Response to Reviewers1017.docx [file pone.0337412.s004.docx]

We are pleased and grateful for the opportunity to submit our work to your esteemed journal and for the recognition it has received from the Editor and reviewers. We also thank the Editor for the time and effort devoted to the review of our manuscript. In response to the most recent comments, we provide the following point-by-point replies and have made targeted revisions accordingly.

1. Some sentences, especially in the Methodology and Discussion sections, can be simplified or broken into smaller parts for better readability and flow.

**[Response]**

We appreciate your valuable suggestions, which have clearly pointed out areas for improvement in our paper.

**[Changes made]**

In response to the Editor’s comment, we have revised the manuscript throughout by splitting overly long and complex sentences to improve clarity and flow, particularly in the Methodology and Discussion sections. We also replaced several informal expressions with standard academic wording to enhance readability.

2. A few technical terms like "multi-dilation atrous branches" and "boundary localization" could benefit from more intuitive explanations or additional context for readers less familiar with the specifics.

**[Response]**

Thank you for your comments, which provide constructive guidance on improving the manuscript’s readability and will help the paper better serve a diverse readership.

**[Changes made]**

After the two terms noted by the Editor, we added:

“parallel dilated convolution branches with different dilation rates (multi-dilation atrous branches)” (see line 138).

“Together, DSDF and T-PACE enhance boundary localization—i.e., precise edge delineation for vegetated and ancient landslides—while preserving robustness for new landslides.” (see line 140).

Following the cross-attention (MHCA) module, we added:

“Cross-attention here can be read as letting RGB ‘ask questions’ and the DEM ‘provide context’, so high-level RGB features retrieve terrain cues that are consistent with large-scale geomorphology.” (see lines 234–236).

We further clarified the FPN description as:

“a lightweight Feature Pyramid Network (FPN) decoder head, a standard module for multi-scale feature fusion, to generate the final two-channel mask.” (see line 147).

We also explained the term “logits” as:

“logits (the raw scores before converting to class probabilities)” (see line 181).

3. Ensure that all figures and tables are referenced properly and are clearly labeled, which will help readers interpret the results more easily

**[Response]**

We thank the Academic Editor for the helpful comment. Upon re-examining the manuscript, we confirmed that there are instances of duplicated citations.

**[Changes made]**

In response to the comments, we corrected minor errors in several figures. We also verified that all Figures and Tables are cited in numerical order and in accordance with journal style; specifically, we adjusted their placement so that each item appears in the main text immediately after its first in-text citation. The figures were prepared in Visio and exported at the highest resolution, then converted using PACE as required by PLOS to ensure compliance with the journal’s figure specifications. In addition, we added necessary explanatory details to the figure captions.
